# Robust Ear Recognition via Nonnegative Sparse Representation of Gabor Orientation Information

**DOI:** 10.1155/2014/131605

**Published:** 2014-02-24

**Authors:** Baoqing Zhang, Zhichun Mu, Hui Zeng, Shuang Luo

**Affiliations:** School of Automation and Electrical Engineering, University of Science and Technology Beijing, Beijing 100083, China

## Abstract

Orientation information is critical to the accuracy of ear recognition systems. In this paper, a new feature extraction approach is investigated for ear recognition by using orientation information of Gabor wavelets. The proposed Gabor orientation feature can not only avoid too much redundancy in conventional Gabor feature but also tend to extract more precise orientation information of the ear shape contours. Then, Gabor orientation feature based nonnegative sparse representation classification (Gabor orientation + NSRC) is proposed for ear recognition. Compared with SRC in which the sparse coding coefficients can be negative, the nonnegativity of NSRC conforms to the intuitive notion of combining parts to form a whole and therefore is more consistent with the biological modeling of visual data. Additionally, the use of Gabor orientation features increases the discriminative power of NSRC. Extensive experimental results show that the proposed Gabor orientation feature based nonnegative sparse representation classification paradigm achieves much better recognition performance and is found to be more robust to challenging problems such as pose changes, illumination variations, and ear partial occlusion in real-world applications.

## 1. Introduction

Because of its wide use in many application domains, biometrics has been a hotspot of pattern recognition and modern security technique development in the past few years. Although great research interests have been devoted to face, fingerprint, and iris, it is believed that each biometrics has its own strengths and weaknesses, and no single biometrics can be expected to meet all the requirements imposed by all applications [1]. Therefore, further research efforts are needed to exploit other potential biometrics modalities that can be conveniently acquired at lower cost. The ear is an emerging biometrics modality with rich and stable structure that does not change significantly as an individual grows [2]. And the ear has “desirable properties such as universality, uniqueness, and permanence” [3]. Moreover, the expression problem, which is a challenging and unsolved bottleneck for unconstrained face recognition, does not exist for ear recognition as ears have a wealthy of structural features that resist the expression variations. Additionally, the acquisition of ear images is considered to be nonintrusive by most people in comparison with other biometrics modalities such as fingerprints or iris. Because of these properties, academic interests in ear recognition have grown significantly in the past few years [4, 5].

Using 3D models for recognition is considered as a promising solution to ear identification with pose changes or illumination variations [6, 7]. However, the slow speed of 3D ear data acquisition limits its wide use in civilian scenarios. What is more, the currently employed 3D data acquisition device is not only expensive and large in size but also imposes more restrictions on the subject. These are obviously not suitable for nonintrusive human identification in real applications. Therefore, the focus of our work is to exploit the 2D ear images which can be conveniently acquired using low cost digital camera.

Varieties of approaches have been explored in the literature for ear identification by using 2D ear images. Chang et al. [8] applied classical Principal Component Analysis (PCA) to face and ear recognition separately, and experimental results showed that ear and face did not have much difference on recognition performance. Hurley et al. [9] developed a new method of localizing ear shape features by using force field transform. Arbab-Zavar and Nixon [10] proposed a model-based approach for ear recognition. The proposed method was based on a model derived by a stochastic clustering on a set of scale invariant features extracted from the training set. Kumar and Wu proposed an ear recognition approach by using the phase information of Log-Gabor filters to encode local ear structures. Yuan and Mu [11] proposed a 2D ear recognition approach based on local information fusion to deal with ear recognition under partial occlusion. However, most of these research works focused on ear recognition in constrained environments. Great efforts are still needed to effectively deal with challenging problems such as pose changes, illumination variations, and image occlusion so as to realize robust ear recognition in real-world applications.

Recently, Wright et al. [12] proposed an innovative classification algorithm SRC (sparse representation based classification) for face recognition, which is demonstrated to be especially effective to deal with image occlusion and corruption. The success of SRC boosts the research interests in sparse representation based ear recognition. Later, Naseem et al. [13] directly applied SRC to ear recognition, which is the first time to address ear biometrics in the context of sparse representation. Huang et al. [14] developed a robust face and ear based multimodal biometric system using sparse representation, which integrated the face and the ear at feature level. In the framework of sparse representation based classification listed above, extracted features from the training data are used to develop a dictionary. Then classification is performed by representing the extracted features of the test data as a sparse linear combination of atoms in the dictionary. However, what needs to be pointed out is that the extracted feature is still critical [15] to sparse representation based classification for ear recognition. Since the number of training samples is often limited in real applications, ear features such as Eigen ear and Random ear are not effective to handle the variations of illumination, pose, and local deformation. Therefore, in the framework of sparse representation based classification, significant efforts are required to investigate more discriminative features that are robust to pose changes, illumination variations, or ear occlusion.

The rich texture information contained in structures of the outer ear can be encoded using Gabor filters. Moreover, local Gabor features are less sensitive to variations of illumination or pose than the holistic features such as Eigen ear and Random ear. Therefore, Gabor related research has been explored in ear recognition. Wang and Yuan proposed to extract local frequency features by using Gabor features and general discriminant analysis [16]. Kumar and Zhang [17] used Log-Gabor wavelets to extract the phase information from the gray-level ear signals. Khorsandi et al. [18] presented a fully automated approach for ear recognition using sparse representation of Gabor features from the ear images. All of these methods lead to improved ear recognition performance. However, the redundant conventional Gabor features are defined via concatenation of the magnitude coefficients resulting in extremely high dimensionality of features since Gabor filters with multiple scales and directions are adopted.

This paper investigates an effective algorithm based on nonnegative sparse representation of Gabor orientation feature for ear recognition under pose changes, illumination variations, and image occlusion. Our feature extraction scheme is to construct Gabor orientation features. Compared with conventional Gabor features, the proposed Gabor orientation feature can not only reduce the information redundancy but also effectively describe the ear shape contours. Then, Gabor orientation feature based nonnegative sparse representation classification (Gabor orientation + NSRC) is proposed for ear recognition. The proposed classification algorithm treats the Gabor orientation features from all the training samples as the dictionary of our nonnegative sparse representation classification (NSRC) for ear recognition, and then the test ear is represented as a linear additive (without subtraction) combination of the Gabor orientation features extracted from all the training ear samples. Compared with SRC in which the sparse coding coefficients can be negative, allowing the data to “cancel each other out” by subtraction, the nonnegativity of our Gabor orientation feature based nonnegative sparse representation classification algorithm conforms to the intuitive notion of combining parts to form a whole and therefore is more consistent with the biological modeling of visual data. Extensive experimental results show that the proposed classification paradigm achieves much better recognition performance and is found to be more robust to challenging problems including pose changes, illumination variations, and ear partial occlusion.

## 2. Related Work

### 2.1. Gabor Features

The Gabor filters with direction *u* and scale *v* can be defined as follows [19]:

(1) $$\begin{array}{c} \psi_{u,v}\left(z\right)=\frac{{||k_{u,v}||}^{2}}{\sigma^{2}}e^{\left(-{||k_{u,v}||}^{2}{||z||}^{2}/2\sigma^{2}\right)}\left[e^{ik_{u,v}z}-e^{-\sigma^{2}/2}\right], \end{array}$$

where *z* = (*x*, *y*) denotes the pixel of an image, ||·|| represents the norm operation, and *k*_*u*,*v*_ is defined as *k*_*u*,*v*_ = *k*_*v*_ *e* ^*iϕ*_*u*_^ with *k*_*v*_ = *k*_max⁡_/*f* ^*v*^, *ϕ*_*u*_ = *πu*/4. *k*_max⁡_ is the maximum frequency, and *f* is the spacing factor between kernels in the frequency domain.

The Gabor transformation of an image *I*(*z*) = *I*(*x*, *y*) is the convolution of the image with the Gabor filters:

(2) $$\begin{array}{c} G\left(z,u,v\right)=I\left(z\right)*\psi_{u,v}\left(z\right). \end{array}$$

Then the Gabor filtering coefficients *G*(*z*, *u*, *v*) are a complex number that can be rewritten as

(3) $$\begin{array}{c} G\left(z,u,v\right)=M\left(z,u,v\right)\cdot \exp\left(i\theta \left(z,u,v\right)\right), \end{array}$$

where *M*(*z*, *u*, *v*) is the magnitude and *θ*(*z*, *u*, *v*) is the phase. Magnitude information is known to reflect the variations of local energy in the image. Therefore, in most Gabor based feature extraction research, conventional Gabor features are usually defined via concatenation of the magnitude coefficients [20].

### 2.2. Sparse Representation Based Classification (SRC)

The sparse representation based classification (SRC) algorithm was proposed for robust face identification in [12]. We denote by *D*_*i*_ = [*d*_*i*1_, *d*_*i*2_,…, *d*_*in*_*i*__] ∈ *ℜ* ^*m*×*n*_*i*_^ the set of training samples from the *i*th class. *d*_*ij*_, *j* = 1,2,…, *n*_*i*_, is an *m* dimensional vector obtained by stacking the columns of the *j*th sample of the *i*th class. The sparse representation makes an assumption that the input sample can be represented as a sparse linear combination of all the samples from the same class. Thus for a test sample *y*_0_ from the *i*th class, ideally, *y*_0_ could be well approximated by the linear combination of the all the samples within *D*_*i*_; that is, *y*_0_ = *α*_*i*,1_ *d*_*i*1_ + *α*_*i*,2_ *d*_*i*2_ + ⋯+*α*_*i*,*n*_*i*__ *d*_*in*_*i*__.

Assuming that we have *n* training samples from *k* classes, we define the concatenation of the *n* training samples from all *k* classes by *D* = [*D*_1_, *D*_2_,…, *D*_*k*_] = [*d*_11_, *d*_12_,…, *d*_*kn*_*k*__], where *n* = *n*_1_ + *n*_2_ + ⋯*n*_*k*_. Then the test sample *y*_0_ can be rewritten in terms of all training samples as *y*_0_ = *Dα*, *α* = [0,…,0,*α*_*i*,1_,*α*_*i*,2_,…,*α*_*i*,*n*_*i*__,0,…,0]^*T*^ ∈ *ℜ* ^*n*^.

In SRC without occlusion, the sparse solution of the input sample *y*_0_ is solved via *l*_1_-minimization

(4) $$\begin{array}{c} \alpha^{\prime }=\text{arg min}\;{||\alpha ||}_{1}\;\text{subject}\;\text{to}\;y_{0}=D\alpha . \end{array}$$

Then classification is made by

(5) $$\begin{array}{c} \text{identity}\left(y_{0}\right)=\arg\underset{i}{\min}\;r_{i}\left(y_{0}\right)={||y_{0}-D\delta_{i}(\alpha^{\prime })||}_{2}, \end{array}$$

where *δ*_*i*_(*α*′) ∈ *ℜ* ^*n*^ is a new vector whose only nonzero entries are those that are associated with class *i*.

While for image with occlusion or corruption, the occluded or corrupted test sample *y* is rewritten as

(6) $$\begin{array}{c} y=y_{0}+e_{0}=D\alpha +e_{0}=\left[D,D_{e}\right]\left[\begin{array}{c} \alpha \\ \alpha_{e} \end{array}\right]=Bw, \end{array}$$

where *B* = [*D*, *D*_*e*_]. The test sample without occlusion or corruption *y*_0_ and the corruption error *e*_0_ have sparse representation over the training sample dictionary *D* and occlusion dictionary *D*_*e*_, respectively. The occlusion dictionary *D*_*e*_ was defined as identity matrix in our work. The sparse coding coefficient *w* could be solved via *l*_1_-minimization and classification is then made by

(7) $$\begin{array}{c} \text{identity}\left(y\right)=\arg\underset{i}{\min}\;r_{i}\left(y\right)={||y-D\delta_{i}(\alpha )-D_{e}\alpha_{e}||}_{2}. \end{array}$$

## 3. Gabor Orientation Feature Based Robust Representation and Classification

### 3.1. Gabor Orientation Feature Extraction

Conventional Gabor feature extraction technique generates redundant features with extremely high dimensionality of the Gabor features. Consequently, extracting Gabor features is computationally intensive, making the features impractical for real-time application. What is more, redundant information in conventional Gabor features will decrease the discriminative classification capability of features.

In this paper, we extract Gabor orientation information contained in the magnitude of Gabor transform. The input image is firstly convoluted with the Gabor kernel functions to obtain magnitude information across different directions and different scales. Then, for a fixed direction, magnitude information of all the scales at this direction is cumulated to formulate the orientation feature. In comparison with conventional Gabor features, the Gabor orientation feature can not only reduce the feature dimensionality by a factor of the Gabor scale parameter but also reduce the redundancy between the orientation information across various scales. What is more, the Gabor orientation feature extracted in this way strengthens the orientation information of the ear shape contours, which is critical to ear identification as orientation features are more important to identify an ear.

Gabor kernels with 3 different scales (*v* ∈ {0,1, 2}) and four different directions (*u* ∈ {0,1, 2,3}) are used in this paper. We predefine parameters $f=\sqrt{2}$, *k*_max⁡_ = *π*/2, and *σ* = 2*π*. The whole process of the Gabor orientation feature extraction from the ear image is illustrated in Algorithm 1 below.

**Algorithm 1 (Gabor orientation feature extraction of ear images)**

Considering the Following.

1. *Input*: an ear image *I*(*z*) = *I*(*x*, *y*), Gabor kernel functions *ψ*_*u*,*v*_(*z*), for ∀*u*, *v*.
2. Convoluting the ear image with Gabor kernel functions: *G*(*z*, *u*, *v*) = *I*(*z*)∗*ψ*_*u*,*v*_(*z*) = *M*(*z*, *u*, *v*) · exp⁡(*iθ*(*z*, *u*, *v*)).
3. Cumulating magnitude information of all the scales at each direction: *Gabor* *orientation*(*z*, *u*) = ∑_*v*_ *M*(*z*, *u*, *v*).
4. *Output*: Gabor orientation feature *G* defined as concatenation of *Gabor* *s* *c* *a* *l* *e*(*z*, *u*) across all the four directions: *G* = (*Gabor* *orientation*(*z*, 0); *Gabor* *orientation*(*z*, 1); *Gabor* *orientation*(*z*, 2); *Gabor* *orientation*(*z*, 3)).

**Figure 1:**
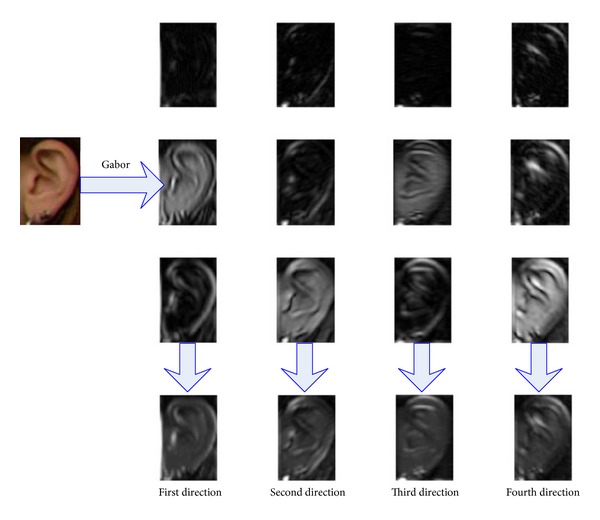
Gabor orientation feature extraction of an ear sample.

Figure 1 illustrates the process of Algorithm 1 to obtain the Gabor orientation features of an input ear image. Obviously, there is a rich amount of redundancies in the filtering responses across different scales and directions. It is easy to see that our proposed Gabor orientation information across four different directions can not only reduce the feature dimensionality by a factor of the Gabor scale parameter but also enhance the orientation information the ear shape contours, which is critical to ear recognition as orientation information is much more important to identify an ear.

### 3.2. Nonnegative Sparse Representation of Gabor Orientation Features

In sparse representation classification described in Section 2.2, the input data is represented as a sparse linear combination of atoms in the dictionary involving both additive and subtractive operations. The negativity of the coding coefficients allows the data to “cancel each other out” by subtraction, which lacks physical interpretation for visual data. In fact, nonnegativity is more consistent with the biological modeling of visual data [21–23] and often produces much better results for data representation [24]. Lee and Seung in [25] argued forcefully for nonnegative representation. Other arguments for nonnegative representation were based on biological modeling, where such constraints are related to the nonnegativity of neural firing rates [26].

Nonnegative sparse representation specializes in that it enforces nonnegativity constraints on the dictionary and sparse coding coefficients; that is, all the elements must be equal to or greater than zero. The nonnegativity constraint leads nonnegative sparse representation to part-based representation. The nonnegative sparse representation model can be formulated as

(8) $$\begin{array}{c} \alpha^{\prime }=\text{arg min}{||\alpha ||}_{1}\;\text{subject}\;\text{to}y_{0}=D\alpha \end{array}$$

with additional constraints ∀*i*, *j* : *D*_*ij*_ ≥ 0, *α*_*i*_ ≥ 0, which is different from standard sparse representation model.

Appropriate dictionary design plays an important role in the framework of sparse representation based classification algorithm [27]. The proposed Gabor orientation feature can not only enhance orientation information of the ear shape but also tolerate image local deformation to some extent. In this paper, we propose to use Gabor orientation features extracted from all the training ear images as the dictionary of our Gabor orientation feature based nonnegative sparse representation classification model.

Supposing there exist *n* training ear images from *k* distinct classes with *n*_*i*_ images from the *i*th class, we firstly extracted Gabor orientation feature from every training ear sample identified with the vector *g*_*ij*_ ∈ *ℜ* ^*m*^, called atoms of our Gabor orientation feature based nonnegative sparse representation classification. Here *i* denotes the index of the class, *i* = 1,2,…, *k*, and *j* denotes the index of the training sample, *j* = 1,2,…, *n*_*i*_. Then, all the atoms from the *i*th class are arranged as columns of a matrix *G*_*i*_ = [*g*_*i*1_, *g*_*i*2_,…, *g*_*in*_*i*__]. Finally, the dictionary of our Gabor orientation feature based nonnegative sparse representation classification is defined as the concatenation of the *n* atoms from all *k* classes: *G* = [*G*_1_, *G*_2_,…, *G*_*k*_] = [*g*_11_, *g*_12_,…, *g*_*kn*_*k*__].

Thus the linear representation of *y*_0*G*_, denoting the Gabor orientation feature of the test ear *y*_0_ from the *i*th class, can be represented in terms of all the atoms in dictionary *G* as *y*_0*G*_ = *Gx*, where *x* = [0,…,0,*α*_*i*,1_,*α*_*i*,2_,…,*α*_*i*,*n*_*i*__,0,…,0]^*T*^ ∈ *ℜ* ^*n*^ is a coefficient vector whose entries are zero except for those from the same class as the test ear signal*y*_0_. *x* is called the sparse coding coefficient.

In SRC, the test data is represented as a combination of atoms in the dictionary involving both additive and subtractive interactions. Here, we propose to express the test ear sample as a linear additive (with nonsubtraction) combination of all the atoms. Our proposed Gabor orientation feature based nonnegative sparse representation classification (Gabor orientation + NSRC) treats Gabor orientation feature as the atoms of the dictionary *G*. According to the feature extraction process presented in Section 3.1, the elements in the dictionary *G* are all nonnegative, so additional constraints only need to be enforced on the sparse coefficient *x*; that is, *x*_*i*_ ≥ 0, for *i* = 1,2,…, *n*. So the proposed Gabor orientation feature based nonnegative sparse representation (GNSRC) model for ear recognition is given below:

(9) $$\begin{array}{c} x_{0}=\text{arg min}\;{||x||}_{0}\;\text{subject}\;\text{to}\;y_{0G}=Gx,\;x_{i}\geq 0. \end{array}$$

Similar to SRC, after the nonnegative sparse coding coefficient *x* is computed by algorithm given in section below, classification is then made by

(10) $$\begin{array}{c} \text{identity}\left(y_{0}\right)=\arg\underset{i}{\min}r_{i}\left(y_{0G}\right)={||y_{0G}-G\delta_{i}(x_{0})||}_{2}, \end{array}$$

where *δ*_*i*_(*x*_0_) ∈ *ℜ* ^*n*^ is a new vector whose only nonnegative entries are those that are associated with class *i*.

When the test ear image is occluded, the test ear sample is rewritten as

(11) $$\begin{array}{c} y_{G}={y_{0}}_{G}+e_{0}=Gx_{0}+e_{0}=\left[G,I\right]\left[\begin{array}{c} x_{0}\\ e_{0} \end{array}\right]=\bar{G}w_{0}, \end{array}$$

where *w*_0__*i*_ ≥ 0, for *i* = 1,2,…, *n*,…, *n* + size(*I*).

Then classification strategy in (10) should be modified as

(12) $$\begin{array}{c} \text{identity}\left(y\right)=\arg\underset{i}{\min}\;r_{i}\left(y_{G}\right)={||y_{G}-e_{0}-G\delta_{i}(x_{0})||}_{2}. \end{array}$$

The solution of our Gabor orientation feature based nonnegative sparse representation classification model based ear recognition algorithm is presented in section below.

### 3.3. Nonnegative Sparse Solution to Gabor Orientation Feature Based Nonnegative Sparse Representation Classification

Recent theoretical development in sparse representation reveals that *l* ^1^-minimization can be used to approximate *l* ^0^-minimization problem, making the problem of (9) convex in the dictionary *G* while still encouraging sparse solutions [28]. Moreover, it is suggested that *l* ^1^-minimization leads to more stable active sets and is preferred for the classification tasks [27]. Therefore, we propose to approximate the solution of (9) via *l* ^1^-minimization. By replacing *l* ^0^ with the *l* ^1^ norm, the Gabor orientation feature based nonnegative sparse representation classification algorithm can be modeled as

(13) $$\begin{array}{c} x_{1}=\text{arg min}\;{||x||}_{1}\;\text{subject}\;\text{to}\;y_{G}=Gx \end{array}$$

with constraints *x*_*i*_ ≥ 0, for *i* = 1,2,…, *n*. This can be rewritten as a standard problem in linear optimization under quadratic and linear inequality constraints.

Therefore, for an appropriate Lagrange multiplier *λ* which controls the compromise between accurate reconstruction and sparseness, the solution to the problem (13) is precisely the solution to the unconstrained optimization problem:

(14) $$\begin{array}{c} O_{F}\left(G,x\right)=\frac{1}{2}||y_{G}-Gx||+\lambda \underset{i}{\sum }x_{i}. \end{array}$$

The dictionary *G* is known because atoms of *G* are defined as the Gabor orientation features extracted from all the training ear samples. Therefore (14) is quadratic with respect to *x*. The global minimum can be calculated using optimization algorithms such as gradient descent and quadratic programming. In paper [29], an efficient algorithm was presented to solve (14). The update rule is given below:

(15) $$\begin{array}{c} x_{t+1}=\frac{x_{t}G^{T}y_{G}}{G^{T}Gx_{t}+\lambda M^{T}},\;\;M=\left[1,1,\ldots ,1\right]. \end{array}$$

## 4. Experimental Results

In this section, we will investigate the use of our proposed Gabor orientation feature based nonnegative sparse representation classification to deal with ear recognition under challenging problems such as pose changes, illumination variations, and ear partial occlusion. Extensive experiments are carried out on UND database Collection J2 and USTB ear database to validate the claims of the previous sections.

### 4.1. Robustness to Challenging Problems: Pose Changes and Illumination Variations

**Figure 2:**
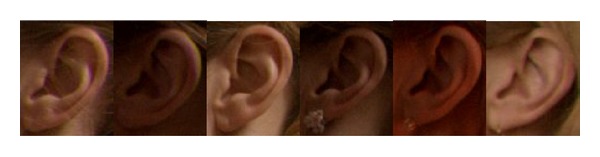
Typical cropped ear images from one subject.

UND database Collection J2 [30] is used in the experiment to evaluate the performance of our proposed algorithm for ear recognition under varying poses and illuminations. Established between 2003 and 2005, UND database Collection J2 consists of 415 subjects. Some of the ear images undergo obvious pose changes or illumination variations, and some are with occlusion. An improved Adaboost algorithm is used to detect and locate ear area automatically [31]. Typical cropped ear images from one subject are shown in Figure 2. It is obvious to see that ear samples in this database suffer from large pose changes and illumination variations.

**Figure 3:**
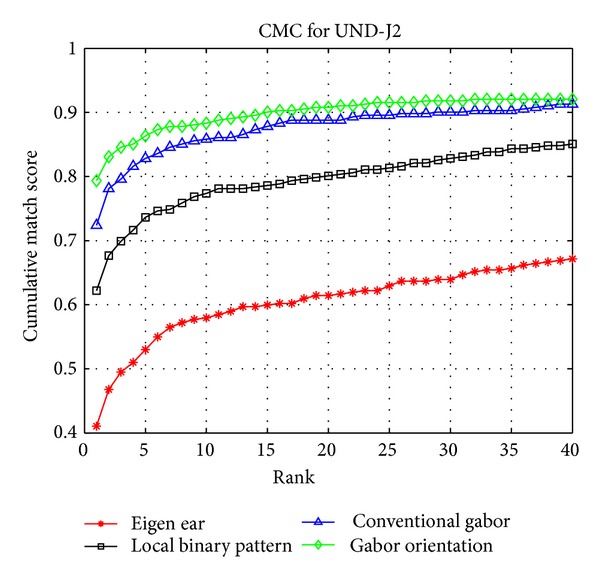
The CMC curves of rank 40 from the recognition experiments on UND ear database.

In this experiment, we randomly select one ear image per subject for testing and the remaining ear images are used as training samples. No preprocessing operations such as denoising, illumination normalization, or pose normalization are carried out in this experiment. We compare the recognition performance of our proposed Gabor orientation feature with Eigen-ear, LBP (Local Binary Patterns), and conventional Gabor features using nearest neighbor (NN) as classifier.  Figure 3 illustrates the cumulative match characteristics (CMC) curves of these four ear recognition approaches. The experimental results illustrate that our proposed Gabor orientation feature outperforms conventional Gabor feature, LBP, and Eigen-ear even using simple NN classifier for recognition. It demonstrates that our proposed Gabor orientation feature is effective to describe ear features.

In order to investigate the recognition performance of our proposed Gabor orientation feature based nonnegative sparse representation classification (Gabor orientation + NSRC), the number of the training samples is required to be sufficiently large to accurately determine the identity of the test sample. Nevertheless, some subjects only contain a few images (2 or 4 images) in this database. These subjects are definitely not suitable for sparse representation classification. We choose subjects that have more than 10 images and a total of 60 subjects meet this criterion. We randomly select five images each subject for testing, the remaining images are selected for training. In the framework of sparse representation based classification algorithm, the dimensionality of the atoms in the dictionary will greatly affect the running time of recognition. Therefore, after conventional Gabor feature and our proposed Gabor orientation features are extracted, the features are subsequently downsampled to 60, 120, 240, 360, 480, 600, 720, and 840.

**Figure 4:**
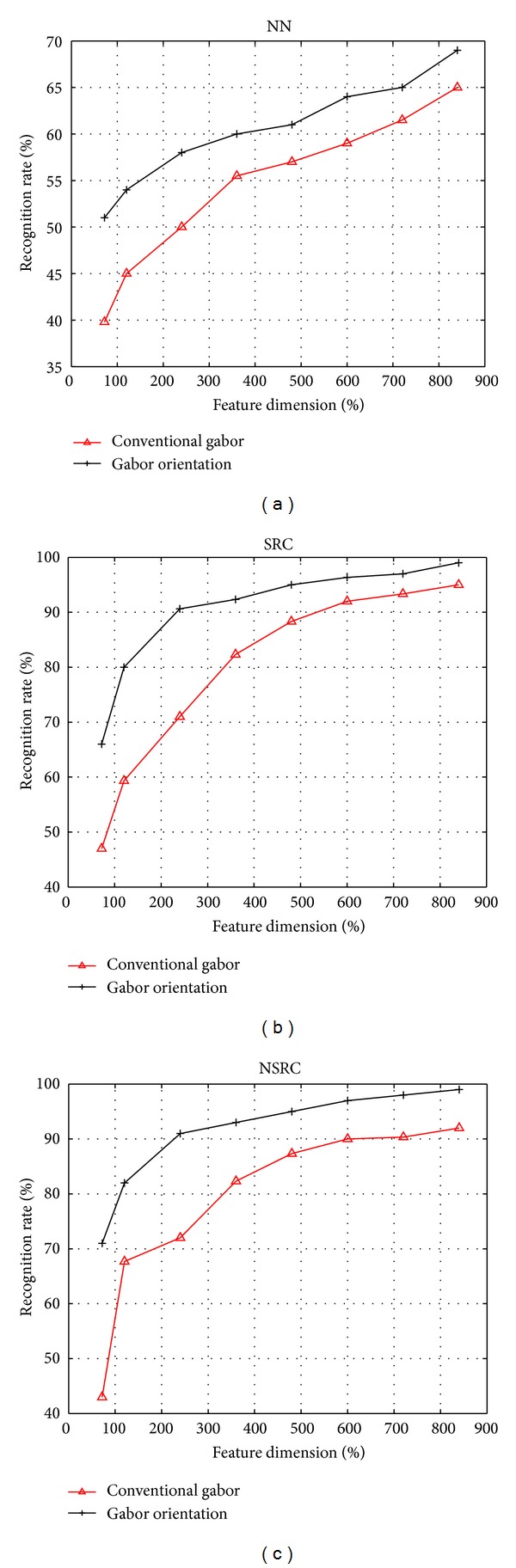
Recognition rates for our proposed Gabor orientation features and conventional Gabor features using various classifiers: (a) NN, (b) SRC, and (c) NSRC (our proposed classifier).

The rank one recognition performances of conventional Gabor features and our proposed Gabor orientation features using NN, SRC, and NSRC under different feature dimensionalities are illustrated in Figure 4. As can be seen in Figure 4, when using the same classifier for classification, our proposed Gabor orientation feature achieves much better performance than conventional Gabor feature. That is because, in comparison with conventional Gabor features, our proposed Gabor orientation features can not only reduce the redundancy between the orientation information across different scales but also enhance the orientation information of the ear shape contours. It is easy to see that the proposed Gabor orientation + NSRC achieves the best recognition performance on UND ear database J2 with large pose changes and illumination variations.

Table 1 lists the recognition performance comparisons of our proposed Gabor orientation feature using different classifiers: NN, SRC, and NSRC. As can be seen in Table 1, our proposed Gabor orientation feature based nonnegative sparse representation classification (Gabor orientation + NSRC) achieves the best performance. When the feature dimension exceeds or equal to 240, it acquires a recognition rate of more than 90%, which is a break-taking recognition performance on such a database under challenging practical conditions including pose changes and ambient illumination variations. That is because the nonnegativity of the proposed Gabor orientation feature based nonnegative sparse representation classification algorithm is more consistent with the biological modeling of visual data and therefore leads to an improved recognition performance.

From the experimental results given in Figure 4 and Table 1, we can conclude that the proposed Gabor orientation feature based nonnegative sparse representation classification algorithm is effective to deal with ear recognition under challenging conditions with varying poses and illuminations.

### 4.2. Robustness to Occlusion

Ear occlusion is considered as a challenging problem inevitable in real applications as ears are often occluded by some objects including hair, hat, or earring [6]. Occlusion poses a great obstacle to robust ear identification in real-world application scenarios. As a result, ear recognition with partial occlusion is addressed as an open challenging problem in most ear recognition related researches [1, 7]. In this section, we will specialize in evaluating the robustness of our proposed Gabor orientation feature based nonnegative sparse representation classification algorithm (Gabor orientation + NSRC) for ear recognition under random occlusion.

**Figure 5:**
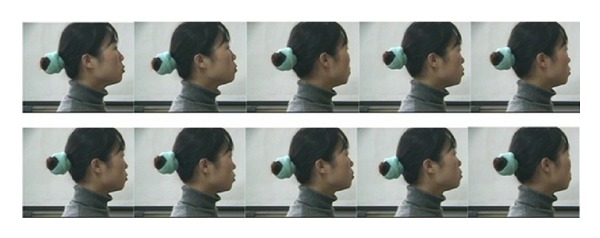
A typical subject from USTB database III under pose variations.

Most of the available ear databases suffer from pose changes, illumination variations, and partial occlusion simultaneously. Our USTB ear database III [32] is publicly available for academic research. On our USTB ear database III, all images are acquired with color CCD camera under the white background and constant lighting. Furthermore, a total of 20 ear images are acquired for each subject, sufficient for the sparse representation based classification algorithm. Because of these properties, this database is suitable for carrying out specialized research on ear recognition with partial occlusion, excluding other influencing factors such as illumination variations and pose changes.  Figure 5 presents a typical subject from this ear database.

**Figure 6:**
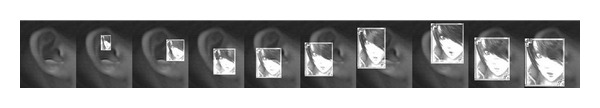
Occluded test ear images from USTB ear database III.

We randomly occlude the test ear image with 5, 10, 15, 20, 25, 30, 35, 40, and 50 percent by replacing a block of each test ear image with an unrelated image to evaluate our proposed Gabor orientation feature based nonnegative sparse representation classification (Gabor orientation + NSRC) for ear recognition under various levels of random occlusion. The location of occlusion is randomly chosen and is unknown to the computer, which is rational in real-world ear recognition applications. We choose interval of 5 because ear is smaller in size and small increase in occlusion range can have severe impact on recognition performance in comparison with face images. In our work, various levels of occlusion at any locations are evaluated more thoroughly to demonstrate the effectiveness of the proposed algorithm for ear recognition under occlusion.  Figure 6 illustrates the randomly occluded test ear images.

**Figure 7:**
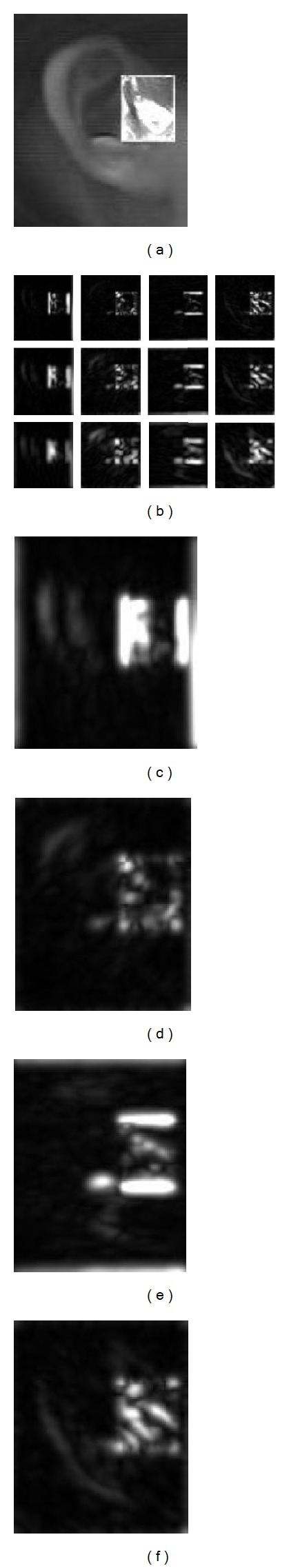
The process of Gabor orientation feature extraction from a randomly occluded test ear sample: (a) a 25% occluded test ear image from the first class of USTB ear database III. (b) Gabor filtering responses of the input ear sample; (c), (d), (e), and (f) present the Gabor orientation information across four different directions.

**Figure 8:**
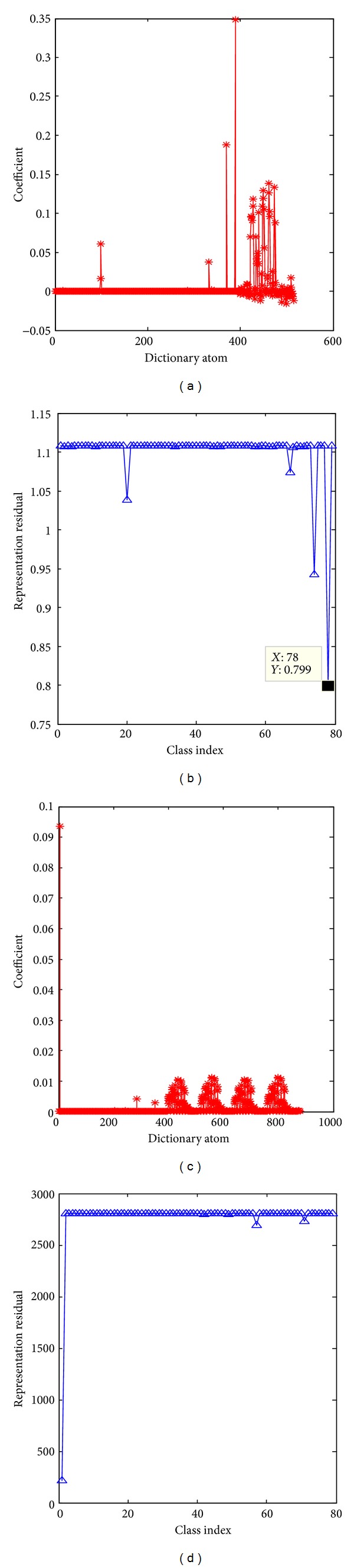
Sparse coding coefficients and representation residual of the 25% occluded test ear image from the first class of USTB; (a) and (c) plot the coding coefficients of Gabor orientation + SRC and Gabor orientation + NSRC. (b) and (d) illustrate the representation residual associated with each class by Gabor orientation + SRC and Gabor orientation + SRC, respectively.

The whole process of Gabor orientation feature based sparse representation classification for an occluded test ear image is illustrated in Figures 7 and 8. The feature extraction process of the proposed Gabor orientation feature based sparse representation classification is illustrated in Figure 7.  Figure 7(a) shows a 25% randomly occluded test ear sample from the first class of USTB ear database III.  Figure 7(b) shows the Gabor filtering responses of the occluded test ear sample. Figures 7(c), 7(d), 7(e), and 7(f) illustrate the Gabor orientation information of the test ear sample across four different directions.  Figure 8 illustrates the classification process of the proposed Gabor orientation feature based sparse representation classification algorithm. Figures 8(a) and 8(b) plot the sparse coding coefficients and representation residual using Gabor orientation feature based sparse representation classification (Gabor orientation + SRC). Figures 8(c) and 8(d) plot the nonnegative sparse coding coefficients and representation residual using Gabor orientation feature based nonnegative sparse representation classification (Gabor orientation + NSRC). We see that Gabor orientation + NSRC correctly classifies the 25% occluded test ear to the first class of the database. However, the occluded ear sample is wrongly classified using Gabor orientation + SRC. Although the representation coefficients are both sparse for Gabor orientation + SRC and Gabor orientation + NSRC, the main difference lies in that the representation coefficients of our proposed Gabor orientation + NSRC are all nonnegative. It demonstrates that nonnegativity is more consistent with the biological modeling of visual data and our Gabor orientation + NSRC exhibits greater robustness to ear image occlusion compared with Gabor orientation + SRC.

**Figure 9:**
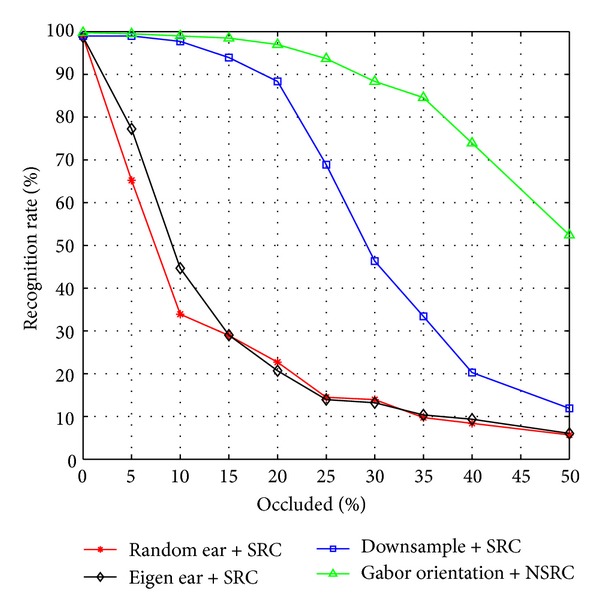
Ear recognition rates on USTB ear database III with various levels of random occlusion.

The recognition rates when the ear is occluded using Gabor orientation + NSRC, Downsample + SRC, Random ear + SRC, and Eigen ear + SRC are illustrated in Figure 9. From the results described in Figure 9, we can see that our Gabor orientation feature based sparse representation classification (Gabor orientation + NSRC) realizes the best recognition performance. Even when the occlusion percent reaches 25%, the proposed Gabor orientation + NSRC algorithm can still achieve a recognition rate of more than 90%, greatly surpassing the other three methods. With the occlusion percent becoming larger, the advantage of our proposed Gabor orientation + NSRC over other three approaches is getting higher. That is because, compared with other three feature extraction methods, the proposed Gabor orientation feature can effectively encode more precise orientation information of ear shape contours, which is more robust to ear image local deformation to some extent. Furthermore, the nonnegativity of Gabor orientation + NSRC conforms to human visual perception and therefore leads to better recognition performance. In a word, the proposed Gabor orientation feature based sparse representation classification shows greater robustness to ear partial occlusion and achieves a promising performance for ear recognition under random occlusion.

Table 2 lists the direct recognition performance comparisons between conventional Gabor feature and our proposed Gabor orientation feature under the framework of sparse representation based classification. As can be seen in Table 2, for the same Gabor orientation feature proposed in the paper, Gabor orientation + NSRC outperforms Gabor orientation + SRC greatly, especially when the occlusion percent surpasses 15%. The same phenomenon holds for conventional Gabor feature; that is, conventional Gabor + NSRC surpasses conventional Gabor + SRC greatly, especially when the occlusion percent surpasses 10%. It demonstrates that nonnegativity conforms to the intuitive notion of combining parts to form a whole and hence leads to improved performance for occluded ear recognition. Obviously, the proposed Gabor orientation + NSRC algorithm achieves the best recognition performance and shows great robustness to ear occlusion. Even when the ear occlusion percent reaches 30%, the proposed Gabor orientation + NSRC can still achieve a recognition rate of 88.35%, while none of the other three approaches achieves 50%. The experimental results listed in Table 2 demonstrate that the proposed Gabor orientation feature based nonnegative sparse representation classification algorithm exhibits more robustness to ear occlusion, especially for large scale occlusion.

## 5. Conclusions

In this paper, a new feature extraction approach is proposed by using orientation information of Gabor wavelets. The new Gabor orientation feature extracts orientation information of the ear across different directions and effectively describe the ear shape contour information. Then, combining visual perception characteristics of Gabor orientation features and nonnegative sparse representation, we propose to use Gabor orientation feature based nonnegativity sparse representation classification (Gabor orientation + NSRC) for ear recognition under challenging problems such as pose changes, illumination variations, and ear occlusion. Extensive experimental results on UND J2 ear database and USTB ear database demonstrate the effectiveness of our proposed Gabor orientation features and its superiority over conventional Gabor feature. Especially, when combined with nonnegative sparse representation classification (NSRC), the proposed Gabor orientation feature based nonnegative sparse representation classification algorithm achieves better recognition performance and shows greater robustness to pose changes, illumination variations, and occlusion, which are challenging problems for ear recognition in real applications.

## Figures and Tables

**Table 1:** The rank one recognition rate for the proposed Gabor orientation feature using three different classifiers.

Classifiers	Dimension							
	60	120	240	360	480	600	720	840
Gabor orientation + NN	51	54	58	60	61	64	65	69
Gabor orientation + SRC	66	80	90	92	95	96	97	98
Gabor orientation + NSRC	71	82	91	93	95	97	98	99

**Table 2:** Recognition performance comparisons between conventional Gabor feature and the proposed Gabor orientation using SRC AND NSRC.

Occlusion ratio	0%	5%	10%	15%	20%	25%	30%	35%	40%	50%
Conventional Gabor + SRC	99.49	94.33	64.56	35.19	18.71	10.13	6.33	4.80	3.8	3.8
Conventional Gabor + NSRC	99.75	95.19	83.92	75.95	74.68	58.48	47.59	37.72	29.37	17.7
Gabor orientation + SRC	99.75	99.24	94.68	78.99	54.68	28.35	15.19	9.37	6.33	4.05
Gabor orientation + NSRC	**99.75**	**99.49**	**98.99**	**98.48**	**96.96**	**93.67**	**88.35**	**84.56**	**73.92**	**52.41**
